# Immunofluorometric quantitation and histochemical localisation of kallikrein 6 protein in ovarian cancer tissue: a new independent unfavourable prognostic biomarker

**DOI:** 10.1038/sj.bjc.6600533

**Published:** 2002-09-23

**Authors:** B R Hoffman, D Katsaros, A Scorilas, P Diamandis, S Fracchioli, I A Rigault de la Longrais, T Colgan, M Puopolo, G Giardina, M Massobrio, E P Diamandis

**Affiliations:** Department of Pathology and Laboratory Medicine, Mount Sinai Hospital, Toronto, ON, M5G 1X5, Canada; Department of Laboratory Medicine and Pathobiology, University of Toronto, Toronto, ON, M5G 1L5, Canada; Department of Gynecology, Gynecologic Oncology Unit, University of Turin, Turin, Italy 10126; National Center of Scientific Research ‘Demokritos’, IPC, Athens, 153 10 Greece; Department of Breast and Gynecologic Oncology, S. Anna Hospital, Turin, Italy 10126

**Keywords:** kallikrein 6, prognosis, ovarian cancer, survival, biomarker

## Abstract

Human kallikrein 6 protein is a newly discovered human kallikrein. We determined the amount of human kallikrein 6 in extracts of 182 ovarian tumours and correlated specific activity (ng hK6 mg^−1^ total protein) with clinicopathological variables documented at the time of surgical excision and with outcome (progression free survival, overall survival) monitored over a median interval of 62 months. Thirty per cent of the tumours were positive for human kallikrein 6 (>35 ng hK6 mg^−1^ total protein). Human kallikrein 6-specific immunohistochemical staining of four ovarian tissues that included benign, borderline and malignant lesions indicated a cytoplasmic location of human kallikrein 6 in tumour cells of epithelial origin, although the intensity of staining was variable. Tumour human kallikrein 6 (ng hK6 mg^−1^ total protein) was higher in late stage disease, serous histotype, residual tumour >1 cm and suboptimal debulking (>1 cm) (*P*<0.05). Univariate analysis revealed that patients with tumour human kallikrein 6 positive specific activity were more likely to suffer progressive disease and to die (hazard ratio 1.71 (*P*=0.015) and 1.88 (*P*=0.022), respectively). Survival curves demonstrated the same (*P*=0.013 and 0.019, respectively). Multivariate analysis revealed that human kallikrein 6 positivity was retained as an independent prognostic variable in several subgroups of patients, namely those with (low) grade I and II tumours (hazard ratio progression free survival 4.3 (*P*=0.027) and overall survival 4.1 (*P*=0.023)) and those with optimal debulking (hazard ratio progression free survival 3.8 (*P*=0.019) and overall survival 5.6 (*P*=0.011)). We conclude that tumour kallikrein 6 protein levels have utility as an independent adverse prognostic marker in a subgroup of ovarian cancer patients with otherwise apparently good prognosis.

*British Journal of Cancer* (2002) **87**, 763–771. doi:10.1038/sj.bjc.6600533 www.bjcancer.com

© 2002 Cancer Research UK

## 

Human kallikrein 6 protein (hK6) is one of the newly discovered members of the human kallikrein gene family, a subgroup of 15 serine proteases mapping in tandem to chromosome 19q 13.3–13.4 (Diamandis *et al*, 2000a; Yousef and Diamandis, 2001). Previously called zyme (Little *et al*, 1997), protease M (Anisowicz *et al*, 1996), and neurosin (Yamashiro *et al*, 1997), kallikrein 6 is now the accepted name (Diamandis *et al*, 2000b). hK6 is a trypsin-like serine protease of unknown physiologic function that is secreted as a 223 amino acid protein. hK6 has been found in various biological fluids including milk from lactating women, nipple aspirate fluid, breast cyst fluid, male and female serum, seminal plasma, and amniotic fluid (Diamandis *et al*, 2000c). Various tissues express the hK6 gene and synthesize the protein (Diamandis *et al*, 2000c; Yousef *et al*, 1999). Expression is particularly prominent in the brain (Yamashiro *et al*, 1997), and this, along with the amyloidogenic potential of hK6 (Little *et al*, 1997) has raised the possibility of a role for hK6 in amyloid precursor processing and the development of Alzheimer's disease. Recently, Diamandis *et al* (2000d) have shown that hK6 is elevated in the cerebrospinal fluid and blood of individuals with histologically confirmed Alzheimer's, thereby suggesting hK6 may be of value in the diagnosis of this disorder.

The newly identified members of the kallikrein family are being intensively investigated for their utility as cancer biomarkers, in part because other previously identified members, namely prostate specific antigen (human kallikrein 3) (Diamandis, 1999) and human glandular kallikrein 2 (Rittenhouse *et al*, 1998), have been found to be particularly useful in this regard and in part because it is reasonable to postulate on biological grounds that proteases are important mediators of tumour invasion and metastasis (Woodhouse *et al*, 1997; Kleiner and Stetler-Stevenson, 1999; Matrisian, 1999). Investigations are proceeding as sensitive and specific assays measuring the gene expression or protein mass of each newly identified member of the kallikrein family become available. Tissue expression of the kallikreins examined to date appears to be down-regulated in aggressive forms of breast cancer (hK3 (PSA) (Levesque *et al*, 1998), hK10 (Liu *et al*, 1996; Goyal *et al*, 1998), hK13 (Yousef *et al*, 2000) and hK6 (Anisowicz *et al*, 1996)) and up-regulated in ovarian cancer (hK4 (Obiezu *et al*, 2002), hK10 (Luo *et al*, 2001), hK5 (Kim *et al*, 2001), hK8 (Magklara *et al*, 2001) and hK6 (Tanimoto *et al*, 2001)).

Ovarian cancer is the disease that causes more deaths than any other cancer of the female reproductive system (Riman *et al*, 1998). Current serological markers such as CA125 (Maggino and Gadducci, 2000) and inhibin (Lambet-Messerlian, 2000) have not gained wide clinical acceptance for early diagnosis, and there is continued interest in identifying biomarkers that would facilitate diagnosis of ovarian carcinoma in its early stages or provide insight regarding prognosis of established disease (Menon and Jacobs, 2000). Two recently published studies (Diamandis *et al*, 2000e; Tanimoto *et al*, 2001) have found initial evidence that hK6 may have clinical utility in ovarian cancer. Data from the former group show that significant elevation of hK6 concentration in the serum occurs almost exclusively in individuals with ovarian cancer. Specifically, they found that 66% of 80 individuals with widespread ovarian cancer had serum hK6 in excess of 15 μg l^−1^ while only two out of 217 individuals with a wide array of other malignancies with high tumour burden had the same. Serum hK6 concentration correlated poorly with that of CA125 in the individuals with ovarian cancer. The data from Tanimoto *et al* (2001) show that there is increased expression of hK6 transcripts in ovarian tumours. Using quantitative PCR, they screened 44 ovarian tumours and 10 normal ovaries and found that kallilkrein 6 mRNA expression was significantly elevated in the majority of low grade and high grade tumours, but not in normal ovary. These two recent studies have unequivocally established that there is increased expression of hK6 in ovarian cancer, but the prognostic significance of this expression remains unknown. To examine this question, we have determined the amount of hK6 protein per milligram total protein in extracts of 180 ovarian tumours and correlated this with clinicopathological variables documented at the time of surgical excision and with progression free survival and overall survival. We report the findings of this study here along with the immunohistochemical localisation of hK6 in four ovarian neoplasms of varying cell type and malignant potential.

## MATERIALS AND METHODS

### Ovarian cancer patients

One hundred and eighty patients with primary ovarian cancer were included in this study. These patients underwent surgery for ovarian cancer at the Department of Gynecology, University of Turin, Italy. Patient age ranged from 25 to 82 years with a median of 59 years. Clinical and pathological information documented at the time of surgery included clinical stage of the cancer, grade and histology of the tumour, and amount of remaining tumour. Menopausal status was documented and response to chemotherapy monitored. Tumours were staged according to the International Federation of Gynaecology and Obstetrics (FIGO) criteria. Histologic classification was based on the World Health Organization and FIGO recommendations. Of the tumours included in this study, 80 were classified as serous papillary, 32 as undifferentiated, 27 as endometrioid, 13 as mucinous, 14 as clear cell, 10 as mullerian and four as other. The size of the residual tumours ranged from 0 to 9 cm, with a median of 1.1 cm.

Patients were monitored for survival and disease progression (no apparent progression or progression) for a median duration of 62 months (range 1–99 months). Follow-up information was available for 165 of the patients. Ninety-seven (54%) of these relapsed and 61 (34%) died during the course of the follow-up period.

Investigations were carried out in accordance with the ethical standards of the Helsinki Declaration of 1975, as revised in 1983, and were approved by the Institute of Obstetrics and Gynecology, Turin, Italy.

### Preparation of tumour cell extracts

Tumour tissue was frozen in liquid nitrogen immediately after surgery and stored at −80°C until extraction. Twenty to 100 mg of frozen tissue was pulverized on dry ice to a fine powder and added to 10 volumes of extraction buffer (50 mM Tris, pH 8.0, 150 mM NaCl, 5 mM EDTA, 10 g l^−1^ of NP-40 surfactant, 1 mM phenylmethyl sulphonyl fluoride, 1 g l^−1^ of aprotinin, 1 g l^−1^ of leupeptin). The resulting suspension was incubated on ice for 30 min during which time it was vortexed every 10 min. The mixture was then centrifuged at 14 000 r.p.m. at 4°C for 30 min and the supernatant (cell extract) was collected and stored at −80°C until analysis. Protein concentration of the extract was determined with the bicinchoninic acid method, with albumin as standard (Pierce Chemical Co., Rockford, IL, USA).

### Measurement of hK6 in ovarian cell extracts

The concentration of hK6 in tumour cell extract was quantified with a highly sensitive and specific non-competitive immunoassay for hK6 that has been previously described and evaluated in detail (Diamandis *et al*, 2000c). The assay incorporated two hK6-specific polyclonal antibodies, one raised in mouse and the other in rabbit, in a sequential two site immunometric format with time resolved fluorescence detection. Analysis of standards, tumour cell extracts and control pools was carried out in duplicate in 96-well polystyrene microtiter plates with 200 μl of specimen added to the immunoassay. The standard curve using recombinant hK6 protein ranged from 0.5 μg l^−1^ to 200 μg l^−1^. Assay precision was better than 10%. Signal detection and data reduction were performed automatically by the CyberFluor 615 Immunoanalyzer.

### Localisation of hK6 in ovarian tumour specimens by immunohistochemistry

A rabbit polyclonal antibody was raised against hK6 full-size recombinant protein, produced in yeast cells. Immunohistochemical staining for hK6 was performed according to a standard immunoperoxidase method. Briefly, paraffin-embedded tissue sections (4 μm) were fixed and dewaxed. Endogenous peroxidase activity was blocked with 3% aqueous hydrogen peroxide for 15 min. Sections were then treated with 0.4% pepsin at pH 2.0 for 5 min at 42°C and blocked with 20% protein blocker (Signet Labs) for 10 min. The primary antibody was then added at 1 : 400 dilution for 1 h at room temperature. After washing, biotinylated anti-rabbit antibody (Signet) was added, diluted four-fold in antibody dilution buffer (DAKO). Following incubation and washing, streptavidin tagged horseradish peroxidase was added for 30 min at room temperature. After washing, detection was achieved with amino ethyl carbazole (AEC) for 5–10 min. The slides were counterstained with haematoxylin and then mounted with cover slips.

### Statistical analysis

**Figure 1:**
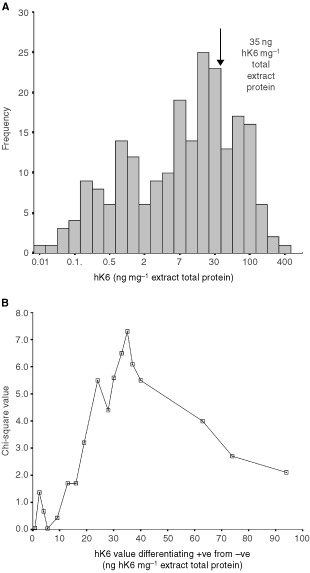
(**A**) Frequency distribution of hK6 specific activity in ovarian tumour extracts. (**B**) Plot of hK6 tumour specific activity *vs* Chi-square statistic to determine the limit between hK6 positive and hK6 negative tumours that is most predictive of overall survival.

Statistical analysis was performed with SPSS software (SPSS Inc. Richmond, CA, USA). To analyse data, patients were divided into different groups according to clinical and pathological parameters. Because the distribution of hK6 mass per mg total protein (i.e. specific activity) in the ovarian tumour extracts was not Gaussian, the nonparametric Mann-Whitney *U*-test was used to determine differences between two groups and the nonparametric Kruskal–Wallis test was used for the analysis of differences among more than two groups. These tests treated hK6 specific activity in the tumour extract (ng hK6 mg^−1^ total protein) as a continuous variable. hK6 tumour extract specific activity was also classified as either hK6-positive (>35 ng mg^−1^ total protein; see Figure 1B

for explanation) or hK6-negative (⩽35 ng mg^−1^ total protein). The relationship of this dichotomous variable to other clinicopathological correlates was established with the Chi Square (χ^2^) test or the Fisher's Exact Test, as appropriate. The impact of tumour extract hK6 specific activity on patient survival and on progression of the disease (progression-free survival) was assessed with the hazards ratio calculated by both univariate and multivariate Cox proportional hazards regression models (Cox, 1972). In the multivariate analysis, the clinical and pathological variables that may affect survival, including stage of disease, tumour grade, residual tumour, histologic type and age were adjusted. Kaplan–Meier progression-free survival and overall survival curves (Kaplan and Meier, 1958) were constructed to demonstrate the survival differences between the hK6-positive and hK6-negative patients. The log rank test (Mantel, 1966) was used to examine the significance of the differences among the survival curves. Following analysis of the entire patient data set as a whole, the process was repeated on subgroups stratified separately by disease stage, by tumour grade and by amount of tumour remaining following surgery (debulking success). The impact of tumour hK6 level (positive or negative) on survival and on disease progression was determined by univariate and multivariate models for each of the subgroups.

## RESULTS

### Distribution of hK6 specific activity in ovarian tumour extracts

The distribution of hK6 specific activity in ovarian tumour extracts from the 180 patients (Figure 1A) ranged from 0.04 ng mg^−1^ total protein to 497 ng mg^−1^ of total protein with a mean of 33 ng mg^−1^ total protein and a median of 13.2 ng mg^−1^ total protein. A value of 35 ng mg^−1^ total protein was identified by Chi square analysis (χ^2^=7.3; *P*=0.007) as the optimal cutpoint to distinguish positive from negative tumours in terms of predicting overall survival (Figure 1B). Thirty per cent of the tumours were hK6 positive by this criterion. hK6 specific activity in tumour extracts was treated both as a continuous variable and as a dichotomous variable (⩽35 ng mg^−1^ total protein=negative, >35 ng mg^−1^ total protein=positive) in the analyses that follow.

**Figure 2:**
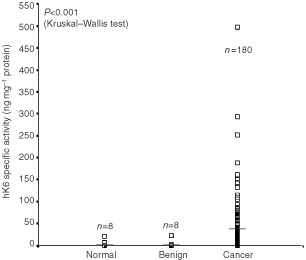
Comparison of hK6 concentration in extracts from normal ovarian tissues (‘normal’), ovarian tissues with benign disease (‘benign’), and ovarian cancer (‘cancer’). *n* indicates the number of specimens in each group. Horizontal bars represent the median hK6 specific activity in each group.

hK6 specific activity (ng hK6 mg^−1^ total protein) was significantly elevated (*P*<0.001 by the Kruskal–Wallis test) in extracts of ovarian tumour (mean 32.7, standard error 3.8, range 0.04 to 497) compared to extracts prepared from normal ovarian tissues (mean 3.5, standard error 2.5, range 0.05 to 20.8) or from ovarian tissue with benign disease (mean 3.2, standard error 2.6, range 0.03 to 21.5) (Figure 2

). Further analysis showed there was no significant difference in hK6 specific activity among the ovarian tumours when they were stratified by histotype (i.e. serous *vs* undifferentiated *vs* endometrioid, etc) (data not shown).

### Relationships between hK6 status and other clinicopathological variables

The distributions of various clinicopathological variables between hK6-positive and hK6-negative patients are summarized in Table 1

**Table 1:**
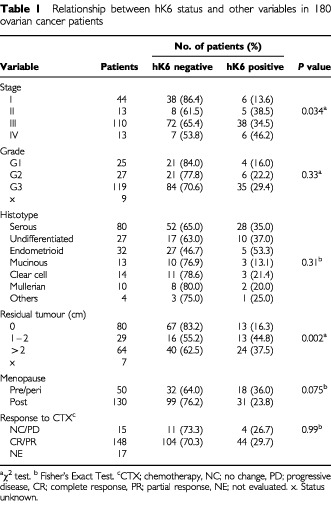
Relationship between hK6 status and other variables in 180 ovarian cancer patients

**Figure 3:**
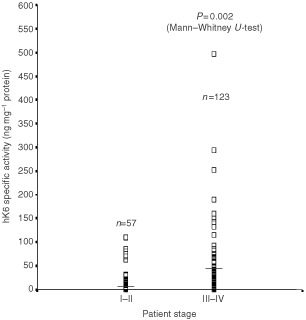
Distribution of hK6 specific activity (ng hK6 mg^−1^ total protein) in tumour extracts from stage I–II and stage III–IV ovarian cancer patients. *n* indicates the number of tumours comprising each group. Horizontal bars represent the median value of hK6 tumour specific activity.

. The relationships between hK6 status and these variables were examined with either the χ^2^ Test or Fisher's Exact Test, as appropriate. No relationship was observed between hK6 status and tumour grade, menopausal status and response to chemotherapy. However, hK6-positive patients were more likely to have advanced disease (stage II–IV), serous tumour histology and greater residual tumour (>1 cm) (all *P*<0.05). hK6 tumour extract specific activity when treated as a continuous variable also associated proportionally with stage of the disease. Figure 3

shows the distribution of hK6 specific activity stratified by disease stage. hK6 specific activity was significantly higher in extracts from stage III–IV ovarian cancer than in those from stage I–II (*P*=0.002 by the Mann–Whitney *U*-Test).

### Univariate and multivariate survival analysis

The impact of hK6 specific activity, other clinicopathological variables and age on disease progression and on overall survival is presented in Table 2

**Table 2:**
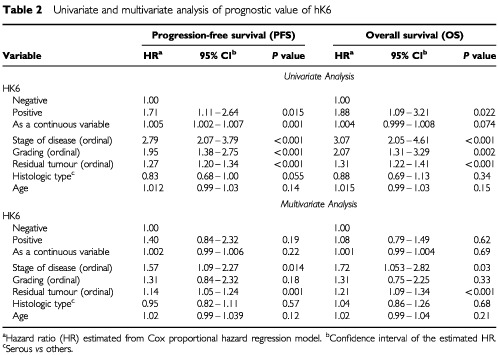
Univariate and multivariate analysis of prognostic value of hK6

**Figure 4:**
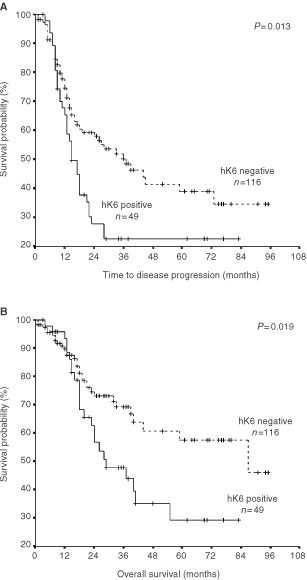
Kaplan–Meier survival curves of the entire patient population under study: Effect of hK6 status. (**A**) Progression-free survival (PFS). (**B**) Overall survival (OS). The adverse effect of hK6 positivity on both time to progression and overall survival was significant.

. In univariate analysis, hK6-positive patients had a significantly increased risk of disease progression (hazard ratio=1.71) and death (hazard ratio=1.88) (*P*<0.05). When hK6 specific activity was treated as a continuous variable, hazard ratios were closely similar to those of hK6 negative tumours (arbitrarily set at 1.00), although the slight increase in risk of disease progression (hazard ratio=1.005) was highly significant at *P*=0.001. Kaplan–Meier survival curves demonstrated survival differences between hK6-positive and hK6-negative patients. As Figure 4

shows, the probability of progression-free and overall survival, respectively, are lower in hK6-positive patients than in hK6-negative patients.

The adverse effects of hK6 positivity on progression free survival and on overall survival were lost in multivariate analysis. As shown in Table 2, when survival outcomes were adjusted for other clinicopathological variables, hK6-positive and hK6-negative patients had statistically similar rates of disease progression and overall survival. Tumour grade also lost its univariate prognostic significance in multivariate analysis. Only stage of disease and residual tumour remaining after surgery maintained their independent effects on survival outcome in the multivariate analysis.

### Univariate and multivariate survival analysis in subgroups of patients

The patients were divided into different subgroups based on disease stage, tumour grade, and debulking success (residual tumour). In each subgroup, the impact of hK6 positivity and negativity on disease progression and on overall survival was determined by univariate and by multivariate Cox proportional hazard regression models. The results are shown in Table 3

**Table 3:**
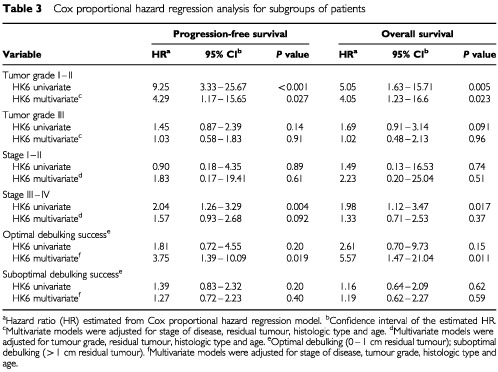
Cox proportional hazard regression analysis for subgroups of patients

**Figure 5:**
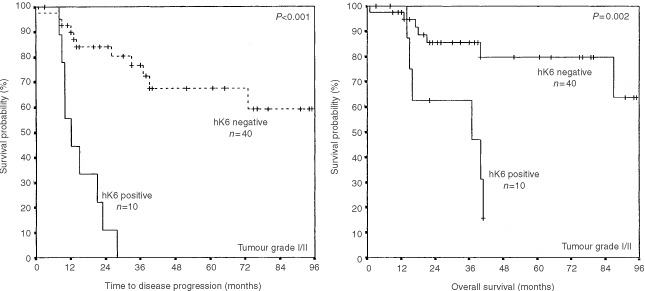
Effect of hK6 status (positive or negative) on progression-free survival (PFS) and on overall survival (OS) among patients with Grade I and II ovarian tumour. The adverse effect of hK6 positivity both on time to progression and on overall survival was significant (*P*⩽0.002).

. hK6 specific activity (positive, negative) significantly impacted survival in the subgroup of 50 patients with tumour grade I or II. Univariate analysis revealed that hK6-positive patients were about nine times more likely to suffer disease progression and five times more likely to die than hK6-negative patients. These survival differences remained significant even after the data were subjected to multivariate analysis. The relative risk of both outcomes arising from hK6 positivity was now about four-fold (*P*<0.03). hK6 status had no such effect among patients with Grade III tumour, nor could any discernible effect be demonstrated among patients with early stage disease and among those with greater than 1 cm of tumour remaining following surgery. Univariate analysis revealed a two-fold increase in risk of disease progression and of death in the subgroup of patients with advanced disease (stage III and IV) who were hK6 positive, but the effect was lost in the multivariate analysis. The opposite occurred in the subset of 80 patients characterised by optimal debulking of the tumour at the time of surgery (remaining tumour less than 1 cm in diameter). hK6 positivity had no demonstrable adverse effect on disease progression or on survival by univariate analysis, but did become statistically significant, giving a 3.5 and 5.5-fold increase in adverse risk, respectively, when the data were subjected to multivariate analysis. The emergence of effects in the multivariate model when none are generated by the univariate model happens when the adjusted variables have no impact at all on the outcome. In the case here, this means that stage of disease, tumour grade, tumour histology and patient age had no prognostic potential on disease progression and overall survival in this particular subset of patients. Kaplan–Meier survival curves of the subset of patients with grade I or II ovarian tumour are shown in Figure 5

. As expected from the univariate analysis mentioned earlier, there was a significant difference in disease progression and survival between hK6 positive and hK6 negative patients.

### Immunohistochemical staining of hK6 in ovarian tumours

**Figure 6:**
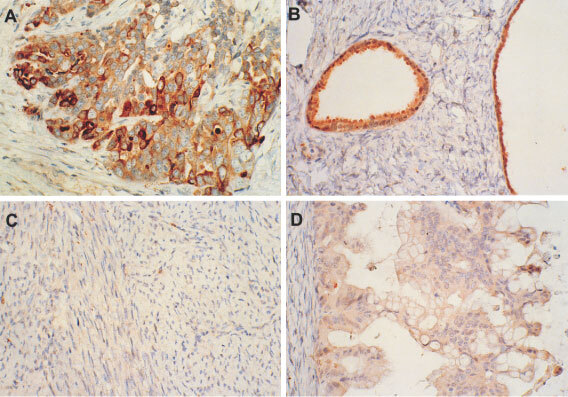
Immunohistochemical localisation of hK6 in ovarian neoplasms of varying malignant potential, cell type and origin (epithelial *vs* mesenchymal). (**A**) Invasive papillary serous adenocarcinoma. (**B**) Serous cystadenofibroma. (**C**) Ovarian leiomyoma, a benign smooth muscle tumour. (**D**) Mucinous epithelial tumour of low malignant potential.

Immunohistochemical staining of hK6 in paraffin embedded tumour sections was roughly proportional to hK6 specific activity in tumour extracts (data not shown). Immunohistochemical localisation of hK6 in ovarian neoplasms of varying malignant potential, cell type and origin (epithelial *vs* mesenchymal) is shown in Figure 6

. Strong cytoplasmic staining of many tumour cells, and absence of any staining of stroma or vessels was observed for the invasive papillary serous adenocarcinoma, the common malignant epithelial tumour of the ovary (Figure 6A). The benign, mixed epithelial and fibrous serous cystadenofibroma presented in Figure 6B stained negligibly in the fibrous component but strongly in the cytoplasm of the epithelium lining the cysts. Staining was negligible for the ovarian leiomyoma, a benign smooth muscle tumour, of Figure 6C, but weakly diffuse throughout the cytoplasm of the neoplastic epithelium of the mucinous epithelial tumour of low malignant potential and intermediate grade depicted in Figure 6D. The supporting stroma (far left) of the latter specimen did not stain. In summary, hK6 staining was restricted to epithelial cells, being absent in mesenchymal elements including fibrous supporting stroma. hK6 stained within the cytoplasm of epithelial cells, but staining intensity was variable among and within tumour preparations.

## DISCUSSION

This is the first report of the prognostic significance of hK6 in ovarian cancer. Increased hK6 synthesis was found to be predictive of more aggressive tumour behaviour over time. Considered apart from other clinicopathological variables and age, hK6 positivity across the entire patient population under study was associated with about a two-fold increase in the risk of both disease progression and of death. This effect was lost when outcomes were adjusted for the other clinicopathological variables and age in multivariate analysis of the entire patient population, but not when the multivariate analysis was restricted to those patients with lower grade tumour and with less residual tumour remaining after surgery (<1 cm in diameter). Among the former subgroup of patients, hK6 positivity predicted about a four-fold increase in the risk of disease progression and of death (*P*<0.03) while corresponding hazard ratios in the latter subgroup were 3.75 and 5.5, respectively (*P*<0.02). The data show that hK6 positivity has independent predictive potential in these two subgroups and gives insight into tumour behaviour over time that cannot be gleaned from the clinical parameters and pathological correlates conventionally measured. Hence hK6 testing could contribute to more individualized effective treatment of such patients.

In accord with recently published work (Tanimoto *et al*, 2001), we found that hK6 is frequently overexpressed in ovarian tumours compared to nonmalignant ovarian tissue. This overexpression tended to be higher in tumour from late stage disease than from early stage disease. Our histochemical studies suggest that hK6 is synthesized by the epithelial cells of the ovary and is distributed diffusely within the cytoplasmic compartment.

Epithelial ovarian cancer has one of the worst prognoses among gynaecologic malignancies, largely because over three-quarters of the diagnoses are made at a time when the disease has already established regional or distant metastases (Gatta *et al*, 1998). Compounding the problem, tumour progression and aggressiveness correlate variably with conventional clinical and pathological markers. Thus there is an important need for additional diagnostic and prognostic markers for this disease and a number of potential markers have been identified. Molecular genetic analysis has uncovered several genes that are altered in a significant fraction of ovarian tumours (Shigemasa *et al*, 1999; Aunoble *et al*, 2000) and has identified other genes that appear to be involved in tumour progression (Suzuki *et al*, 2000). A whole host of serine proteases (Tanimoto *et al*, 1997; Hirahara *et al*, 1998; Underwood *et al*, 1999; Shigemasa *et al*, 2000) in addition to those of the kallikrein family (Tanimoto *et al*, 1999; Diamandis *et al*, 2000a) are overexpressed by epithelial ovarian tumour cells. These may have prognostic potential insofar as they assist in degrading the extracellular barriers such as interstitial connective tissue and basement membrane that must be breached in order for tumour to invade adjacent tissue and metastasize (Duffy, 1992; Aznavoorian *et al*, 1993). Recently, microarray based approaches have been used to assemble multigene expression profiles of neoplastic ovarian tissue. Such studies have identified the gene coding for HE4, a secreted extracellular protease inhibitor, as a promising diagnostic marker (Ono *et al*, 2000) and pinpointed other genes that correlate with serous and mucinous histology (Schummer *et al*, 1999). A recent report (Welsh *et al*, 2001) analysed the expression levels of more than 6000 human genes in 27 ovarian tumours and four normal ovarian tissue samples using this technique. This study identified genes whose expression pattern distinguished between ovarian cancer of low and high malignancy and ranked other genes in terms of the diagnostic potential of their expression. Undoubtedly, the plethora of new candidate molecular markers will lead to insights into the cellular changes that correlate with, or determine the different biological properties and diverse behaviour of, individual ovarian epithelial tumours. The goal for clinical care is to select the battery of molecular markers that are most informative in terms of unmasking the individual biological profile of each tumour, thereby laying out the molecular targets for the most effective therapeutic intervention.

Recently we have found that hK6 is elevated in the serum of a significant number of women with ovarian cancer (Diamandis *et al*, 2000e). Thus, hK6 may have diagnostic potential in addition to its prognostic significance demonstrated in this study. These findings warrant further basic and clinical studies to investigate the role of hK6 in ovarian cancer pathogenesis and progression.
